# Validating a conceptual model for an inter-professional approach to shared decision making: a mixed methods study

**DOI:** 10.1111/j.1365-2753.2010.01515.x

**Published:** 2011-08

**Authors:** France Légaré, Dawn Stacey, Susie Gagnon, Sandy Dunn, Pierre Pluye, Dominick Frosch, Jennifer Kryworuchko, Glyn Elwyn, Marie-Pierre Gagnon, Ian D Graham

**Affiliations:** 1 Tier 2 Canada Research Chair in Implementation of Shared Decision Making in Primary Care, Research Center of the Centre Hospitalier Universitaire de QuébecQuébecCanada and Professor, Department of Family and Emergency Medicine, Université LavalQuébec, Canada; 2 Clinical investigator, Ottawa Hospital Research Institute, Ottawa, Canada and Assistant Professor, Faculty of Health Sciences, School of Nursing, University of OttawaOttawa, Canada; 3 Research Assistant, Knowledge Transfer and Health Technology Assessment, Research Center of the Centre Hospitalier Universitaire de QuébecQuébec, Canada; 4 Student, Ottawa Hospital Research Institute, Ottawa, Canada and Student, Faculty of Health Sciences, School of Nursing, University of OttawaOttawa, Canada; 5 Assistant Professor, Department of Family Medicine, McGill UniversityMontréal, Canada; 6 Assistant Professor, Department of Medicine, University of CaliforniaLos Angeles, CA, USA; 7 Assistant Professor, College of Nursing, University of SaskatchewanSaskatchewan, Canada; 8 Director of Research, Department of Primary Care and Public Health, School of Medicine, Cardiff UniversityCardiff, UK; 9 Assistant Professor, Faculty of Nursing Sciences, Laval University, Québec, Canada and Researcher, Knowledge Transfer and Health Technology Assessment, Research Center of the Centre Hospitalier Universitaire de QuébecQuébec, Canada; 10 Assistant Professor, Faculty of Health Sciences, School of Nursing, University of OttawaOttawaCanada and Vice-President of Knowledge Translation, Canadian Institutes of Health Research, Knowledge Translation PortfolioOttawa, Canada

**Keywords:** conceptual model, decision coaching, inter-professionalism, primary care, shared decision making, validity

## Abstract

**Rationale, aims and objectives:**

Following increased interest in having inter-professional (IP) health care teams engage patients in decision making, we developed a conceptual model for an IP approach to shared decision making (SDM) in primary care. We assessed the validity of the model with stakeholders in Canada.

**Methods:**

In 15 individual interviews and 7 group interviews with 79 stakeholders, we asked them to: (1) propose changes to the IP-SDM model; (2) identify barriers and facilitators to the model's implementation in clinical practice; and (3) assess the model using a theory appraisal questionnaire. We performed a thematic analysis of the transcripts and a descriptive analysis of the questionnaires.

**Results:**

Stakeholders suggested placing the patient at its centre; extending the concept of family to include significant others; clarifying outcomes; highlighting the concept of time; merging the micro, meso and macro levels in one figure; and recognizing the influence of the environment and emotions. The most common barriers identified were time constraints, insufficient resources and an imbalance of power among health professionals. The most common facilitators were education and training in inter-professionalism and SDM, motivation to achieve an IP approach to SDM, and mutual knowledge and understanding of disciplinary roles. Most stakeholders considered that the concepts and relationships between the concepts were clear and rated the model as logical, testable, having clear schematic representation, and being relevant to inter-professional collaboration, SDM and primary care.

**Conclusions:**

Stakeholders validated the new IP-SDM model for primary care settings and proposed few modifications. Future research should assess if the model helps implement SDM in IP clinical practice.

## Introduction

Given today's drive to integrate health care services, foster patient-centred care and engage patients as partners in their own care, finding effective ways to involve patients in decision making has become crucial [1]. According to the literature, shared decision making (SDM) is an approach whereby practitioners and patients communicate around decisions, referring to the best available evidence and deliberating upon the consequences of each option [2–4]. In the process, patients' autonomy is respected, patients are helped to establish their values and preferences, and final treatment decisions are reflected through agreement between patients and their practitioner(s) rather than a unilateral decision.

In most Western health care systems, care is increasingly planned and delivered by inter-professional (IP) teams [5–7]. Inter-professionalism refers to the process in which professionals from different disciplines collaborate in an integrated approach to patient care [5,6]. Key elements of IP collaboration include: the engagement of two or more health professionals from different disciplines, a common goal, collaborative relationships, integrated and cohesive care, symmetrical power, shared knowledge, interactions over time, an understanding of each professionals' role, interdependence among professionals, and a supportive organizational environment [5,8].

Given that most primary care decisions are made by the patient and more than one health care professional, SDM models should acknowledge the involvement of multiple players including IP teams [9]. However, most SDM conceptual models are limited to the clinical encounter between a patient and a single doctor[10]. Nonetheless, an IP approach to SDM has the potential to help primary health care teams collaborate in involving patients in decision making and help improve the quality of decisions by fostering integrated health care services and continuous care across health sectors [11]. More use of SDM could increase the quality of care, reduce variations in practice, and close the gap between the care that patients need and want and the care that they actually receive [12].

A conceptual model is an important element of the research process [13]. An IP-SDM conceptual model has the potential to broaden the perspective of SDM researchers beyond the patient–doctor dyad to include an IP approach. The model could also assist researchers interested in IP to focus on the essential elements that patients need as they move through the decision-making process within specific clinical pathways [14,15]. Finally, an IP-SDM model might help health care teams set clear goals for their patients and contribute to the design of medical and health sciences education curricula. Consequently, our team drew on a detailed theory analysis of SDM models [10] and conducted a stepwise consensus-building exercise to develop a new IP-SDM model that health care teams can use to achieve SDM [16].

Briefly, this new IP-SDM model addresses the three levels of health care systems. The model captures the influence of factors at the micro level (individuals), as well as the influence of systemic factors at both the meso level (health care teams within organizations) and the macro level (broader policies and social contexts). At the individual level, the patient presents a health problem that requires a decision. The patient then moves through a structured process to make an informed, preference-sensitive decision while interacting with one or more health care professionals and family members. The model acknowledges the contribution of each person's role and recognizes two particular roles that can be shared among health care professionals on the team: the role of decision coach (a person who supports the patient's involvement in decision making) and the role of first contact person (a person who identifies the health problem and the decision that must be made). Following our development of the model and consistent with our research protocol [17], the present study aimed to explore the validity of the model in the context of primary care.

## Methods

### Participants

Using a snowball strategy [18], we selected participants from the following categories: (1) stakeholders from Canadian organizations that represented health professionals, medical education and the health care policy environment; (2) patients that represented a health consumers' perspective; and (3) clinicians from primary health care teams who were either familiar or unfamiliar with the concepts of inter-professionalism and/or SDM. Using our personal networks and networks of colleagues not directly involved in our project, we intentionally targeted participants from each of these three categories in two Canadian provinces: Québec and Ontario. All participants completed a consent form. There was no financial compensation.

### Data collection

To help participants better understand the proposed IP-SDM model, we produced a short video illustrating an IP-SDM approach. The video depicts a pregnant woman, her husband and an IP team (a doctor and a nurse) making a decision regarding prenatal screening for Down syndrome. The video is based on a scenario that the research team developed from audiotaped consultations of family doctors and pregnant women [19].

Table 1 briefly describes the storyline.

**Table 1:** The video vignette: a clinical example of the IP-SDM model at the individual level

**Step 1.** ‘The patient and the health condition’ or ‘Equipoise’. A pregnant woman accompanied by her husband meets her family doctor for her first prenatal visit. The family doctor indicates that she will need to decide whether or not to have prenatal screening for Down syndrome.
**Step 2.** ‘Exchange of information’ about the options. The nurse provides the pregnant woman with written information on prenatal screening. The health care team of the clinic is aware of this information.
A few weeks later, the pregnant woman and her husband meet the nurse again. The nurse assesses their understanding of the information they were given, corrects any misperceptions and answers their questions. The nurse involves both the woman and her husband in this exchange.
**Step 3.** ‘Values clarification’. The nurse assesses the values of the woman and her husband regarding prenatal screening for Down syndrome by asking them which outcomes are the most important to them.
**Step 4.** ‘Feasibility of the options’. The nurse reviews the feasibility of the options with the couple in light of accessibility and costs.
The nurse informs the family doctor of the woman and her husband's understanding of the information and describes what matters most to each of them. She also confirms that the options that the couple is considering are feasible. The nurse states that the husband has different values from his wife but that both understand each other's point of view and agree to proceed with prenatal screening for Down syndrome using the blood test.
**Step 5** ‘Preferred/actual choice’. The pregnant woman and her husband meet the family doctor for a second time and convey their decision that the woman will undergo prenatal screening test for Down syndrome.
**Step 6** ‘Implementation’. The family doctor completes the requisition for blood work and tells the couple when to expect the results.

IP-SDM, inter-professional shared decision making.

A member of the research team conducted the interviews (individual or group). All group interviews with health care teams took place at their clinic. Individual interviews were either conducted face-to-face or by telephone. We developed a semi-structured interview guide, which was used for both the individual and group interviews. The interviewer began by describing the IP-SDM model and explaining its core concepts and the relational statements linking the concepts. Next, the interviewer presented the video to the participants. The interviewer then asked a series of open-ended questions and asked informants to: (1) suggest changes to the model that could make the model clearer and/or easier to implement; (2) identify barriers and facilitators to the implementation of an inter-professional approach to SDM in clinical practice; and (3) appraise the model using nine criteria that were based on elements known to be important to developing a theory [20,21]. Each criterion was rated on a 7-point Likert scale ranging from strongly disagree (1) to strongly agree (7), with neutral in the middle. Participants also provided basic sociodemographic information about themselves. All interviews (individual and group) were audiotaped and transcribed verbatim.

### Data analysis

For the qualitative data, two research assistants performed thematic data analysis using NVivo Version 8 to collect, organize and analyse the data. When analysing the data, the research assistants were guided by a coding framework based on known barriers and facilitators to the implementation of SDM [22] and on concepts that the literature associated with IP collaboration [5,7,8]. As well, inductive thematic analysis was used if the data suggested a new theme to be added. Reviewers independently coded two interviews using this coding framework and compared their results. After reaching consensus on coding using the framework, they divided the remaining transcripts for analysis. Results were summarized for each level of the health care system (the micro, meso and macro levels). The principal investigators reviewed and validated the results. For quantitative data, the research team performed simple descriptive statistical analyses using the Statistical Analysis System (SAS Institute, Cary, NC, USA), Version 9.1.3.

### Team consensus on a revised IP-SDM model

**Figure 1:**
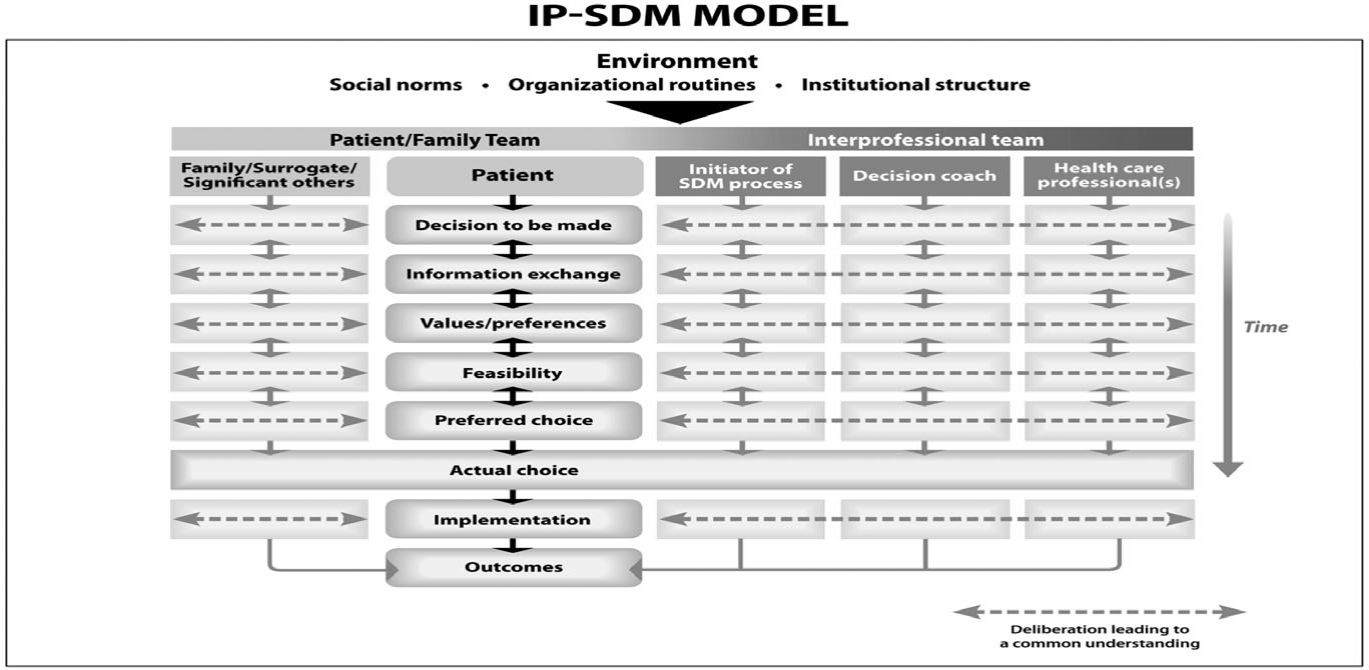
Inter-professional shared decision-making model. IP-SDM, inter-professional shared decision making.

All team members were sent a summary of participants' suggested changes to the initial IP-SDM model. Team members either forwarded their feedback or teleconferenced to discuss the changes and reach consensus on changes required. Finally, a graphic artist helped design the revised model (Fig. 1).

## Results

### Participants

The individual interviews and group interviews were conducted from January to April 2009. Seventy-nine health care professionals and other stakeholders were approached and participated in either an individual interview (*n* = 15) or group interview (*n* = 7). Most group interviews were composed of a diverse set of health care professionals working as a team. Table 2 describes the characteristics of the participants, who represented all levels of the health care system: the micro level (*n* = 63), the meso level (*n* = 6) and the macro level (*n* = 10). The median duration was 83 minutes (SD = 18.7 minutes) for individual interviews and 65 minutes (SD = 12.4 minutes) for group interviews.

**Table 2:** Participants' characteristics and interview modalities

Characteristic	Micro	Meso	Macro	Total
Gender: *n* (%)				
Female	49 (78)	4 (67)	9 (90)	62 (79)
Male	14 (22)	2 (33)	1 (10)	17 (21)
Age: *n* (%)				
Under 30 years	9			9 (11)
30 to 39 years	19			19 (24)
40 to 49 years	14	3	1	18 (23)
50 to 59 years	10	3	7	20 (25)
60 years and older	4		1	5 (6)
Missing data	7		1	8 (10)
Profession: *n* (%)				
Doctor	27			27 (34)
Resident	6			6 (8)
Nurse	8			8 (10)
Clinical nurse	1			1 (1)
Social worker	3			3 (4)
Occupational therapist	1			1 (1)
Pharmacist	1			1 (1)
Audiologist	1			1 (1)
Speech therapist	1			1 (1)
Manager	3	6	10	19 (25)
Patient representative	3			3 (4)
Missing data	8			8 (10)
Interview modality: *n*				
Individual interview	3	6	6	15
Group interview	6	0	1	7

Of 79 participants, 38 (48%) saw the video. The rest could not watch the video because of technical limitations (a screen was not available, interview time was too short). All participants were given a detailed description of the IP-SDM model that has been developed previously by the team.

### Participants' proposals of changes to the model with the research team's response

Participants' suggested changes to the IP-SDM model and the research team decisions to incorporate them are summarized in Table 3. The main changes were to place the patient at the centre of the model, enlarge the concept of family to include significant others, clarify what was meant by outcomes, make the concept of time more explicit, merge the three levels of health care (micro, meso and macro), and explain how two new items influenced the SDM process: emotions and the physical environment. The next paragraphs describe the revised model with attention to the rationale for making the changes.

**Table 3:** Participants' proposed changes to the IP-SDM model and the responses of the research team

		Level			
			
Category	Proposed change	Micro	Macro	Meso	Research team's response
**Actor/role**					
Patient	Make the patient's presence clearer and more central; make explicit that the patient is a decision maker	X	X	X	Patient moved to central position
	Change ‘patient’ to ‘client,’‘consumer’ or ‘person with a health condition’	X	X	X	Retained the term ‘patient’
First contact person	Specify that this role can be played by any health professional involved		X		Added
Decision coach	Make the coaching aspect explicit		X		Added
Family member(s)	Make the concept more inclusive (e.g. include significant others, the patient's social support network, the patient's social network)	X		X	Changed
Health professional(s)	Include non-regulated health care providers: change ‘health care professionals’ to ‘health care providers’ and divide into regulated and non-regulated providers		X		Retained the definition of ‘professional’ selected for the study
**SDM process**					
Decision point situation	‘Equipoise’ is a confusing concept: force the term, change to another concept that is easier to understand, keep ‘decision point’ only or use ‘portrayal of options’	X	X	X	Kept ‘decision to be made’ only
Implementation	Make the box bigger to show that this step takes more time than other steps		X		Made box size consistent throughout the model
Health outcomes	Clarify the type of outcome (patient health outcome versus an outcome related to the IP process). For example, remove the term ‘health’ and add information about what the model means by ‘outcomes’		X		Kept ‘outcomes’ only and expanded description
General modifications	Avoid verbs in labelling the steps. Choose names that are more inclusive and explain names when describing the model		X		Verbs were removed
	Highlight the notion of time to represent the fact that time affects all levels		X		Concept of time was expanded
**Meso/macro level**					
General modifications	Add the meso/macro level as a background to the micro level Add the environment to the micro level		X		The three levels were merged
Environment	Add ‘health professional regulators’ to the environment		X		Not applicable after merging the two figures
	Add the patient and family to the section ‘IP team members’	X			Patient/Family Team added at the same level as inter-professional team
	Represent collaboration between the patient and his/her family or relatives	X			Patient and family moved side-by-side
Additional items	Discuss the relevance of adding the concept of ‘outcomes’ in the meso/macro section			X	Not applicable after merging the two figures
IP team	Mention that the elements are examples and that the list is not exhaustive		X		In accompanying document, mention that ‘health care professionals’ is an inclusive term
**Figures**					
Pyramid	Use bubbles (concentric circles) instead of a pyramid			X	Not applicable after merging the two figures
Arrows	Add feedback loops to represent that IP SDM is not a linear process; discuss the iterative process. State that decisions can be revisited if the results of the first decision fail to meet expectations	X	X	X	Add arrows that represent the iterative process and feedback loop
	Add an arrow or a circle to represent interactions between the health professionals involved in the SDM process Add arrows to represent deliberation between silos	X	X		Added a dotted line between steps of the SDM process
Squares	Represent the steps as circles instead of squares to express the overlap/iterative nature of the process		X		Too difficult to represent graphically
Diamond shapes	Enlarge the diamond shape in the background to clarify that all elements of the model have equal importance		X		Not applicable after merging the two figures
**Missing elements**	Add the physical environment (e.g. the availability of meeting rooms, access to technology)		X		Included in the description of institutional structure
	Add the box ‘Follow-up and revisiting or readjusting if needed’ between ‘Implementation’ and ‘Health outcomes’ or after ‘Health outcomes’	X			Judged unnecessary
	Include a new box to represent affective and emotional aspects, the unconscious dimension of decision making that SDM should take into account	X			Added to the description of Information exchange

IP-SDM, inter-professional shared decision making.

#### The environment

‘Environment’ refers to the global context in which IP-SDM takes place. To illustrate that an IP approach to SDM within clinical encounters is not free of the influence of environmental factors, the top of the revised model (Fig. 1) lists the two interpenetrated categories of meso and macro-level factors: social norms, organizational routines and institutional structure. Social norms include cultural values, routines and policies within society, the health care team, and the patient-family team, all of which influence the decision-making process. In health care organizations, organizational routines are activities that exhibit four characteristics: memory, adaptation, values and rules [23]. Institutional standards are defined as state-level policies that constrain organizations and individuals, including elected officials, government agencies, the public administration, the legislature and the legal system. Neo-institutionalism holds that institutional standards are public supra-organizational, and exist legally for the social good. Examples of institutional standards that impact health decision making are federal, provincial and municipal government rules and policies that constrain resources or legislate requirements for consent; accreditation standards; and practice guidelines set by professional bodies.

#### Actors and their roles

Figure 1 depicts the patient as central to the decision-making process. The initiator of the SDM process plays another central role. The role of initiator can be played by any health care professional – doctor, nurse practitioner, pharmacist – who identifies the health problem and makes explicit the decision to be made. A third key role is the decision coach, who is trained to support the patient's involvement in decision making. The last column of the Fig. 1 refers to health care providers. For the SDM process to be IP, at least two health care providers from different professions must collaborate with the patient either concurrently or sequentially. Finally, the family category (the first column) includes relatives, surrogates and/or other people who are important to the patient and can influence the decision-making process. The family member can support the patient and/or add pressure and make the process more difficult. A surrogate decision maker participates in decision making on behalf of the patient in situations where the patient cannot be involved (for example, if the patient has severe mental illness or is unconscious).

#### Steps in the SDM process

The SDM process begins with the ‘Decision to be made’: the initiator of the SDM process makes explicit that a choice needs to be made and identifies more than one option. We had initially labelled this stage ‘Equipoise,’ which Elwyn *et al*. define as a situation in which more than one option exists (including the option of status quo) and in which the pros and cons of each option must be weighed [24]. We changed the name of this stage to ‘Decision to be made’ after participants found the term ‘equipoise’ too confusing.

The next step in the process is to exchange information about the options (‘Information exchange’). The health professional(s) and the patient share information about potential harms and benefits, including evidence-based information such as educational material and patient decision aids. We expanded this step to include information on the affective and emotional aspects of the decision after participants expressed the opinion that the affective and emotional aspects of the decision-making process may not be explicitly stated but are important to consider.

Participants did not suggest changes regarding the clarification of values and preferences, the feasibility of the options, the preferred choice or the actual choice. They agreed that it was important to acknowledge not only the patient's values and preferences but also to acknowledge the impact of values and preferences of others involved in the decision-making process, including family, surrogates, decision coach, initiator and other health care professionals. They suggested that we modify ‘health outcomes’ for ‘outcomes’ to be more inclusive of other outcomes, which may impact decision making.

#### Interactions between steps in the process and individuals

The iterative nature of the IP-SDM process is represented by the two-way arrows between the steps of the process. These arrows also express the possibility for patients to revisit a decision. Revisiting decisions was considered more likely to occur when an initial choice does not produce the desired health outcome or when chronic conditions are involved (e.g. depression or hot flashes).

### Barriers and facilitators to implementing the IP-SDM model in clinical practice

Table 4 summarizes participants' perceptions of barriers and facilitators to implementing IP-SDM in clinical practice. The three most often reported barriers were time constraints, insufficient resources and an imbalance of power among health professionals. The three most often reported facilitators were education and training in IP and SDM, motivation to achieve an IP approach to SDM, and a mutual knowledge and understanding of the disciplinary roles (the practices, expertise, responsibilities, skills and values). Some barriers and facilitators reported by participants were specific to an IP approach. The barriers most frequently reported were an imbalance of power between health professionals, practicing in silos, and disagreeing about roles and responsibilities. The most frequently reported facilitators in the context of an IP approach to SDM were mutual knowledge and understanding of disciplinary roles, trust and respect.

**Table 4:** Frequency of participants' mention of barriers and facilitators to IP-SPM

Factors	Number of interviews (individual or group) in which the factor was mentioned as a barrier (*n* = 15)	Number of interviews (individual or group) in which the factor was mentioned as a facilitator (*n* = 15)
**1.Knowledge**		
1.1 Unaware/aware of IP-SDM	3	
1.2 Familiar/unfamiliar with IP-SDM	2	
1.3 Lack of education and training/education and training about IP-SDM		11
1.4 Level of knowledge about IP-SDM		1
1.5 Unstandardized/standardized information regarding IP-SDM		1
**2.Attitudes**		
2.1 Lack of agreement/agreement with a specific component of IP-SDM		
2.1.1 Disbelief/belief that IP-SDM is supported by the evidence	1	1
2.1.2 IP-SDM is inapplicable/applicable		
2.1.2.1 Patient characteristics are inappropriate/appropriate for IP-SDM	3	3
2.1.2.2 Clinical situation is inappropriate/appropriate for IP-SDM		3
2.2 Lack of general agreement/general agreement with IP-SDM		
2.2.1 IP-SDM threatens/enhances professional autonomy	2	2
2.2.2 IP-SDM is impractical/practical	1	
2.2.3 IP-SDM is irrelevant/relevant	1	
2.2.4 Overall lack/overall agreement with IP-SDM	1	
2.3 Expectation of difficult feelings/positive feelings from applying IP-SDM		
2.3.1 Patient outcomes will suffer/benefit from IP-SDM	5	3
2.3.2 Health care processes will suffer/benefit from IP-SDM	1	1
2.4 Lack of motivation/motivation to apply IP-SDM	6	7
2.5 Unresponsiveness/responsiveness to using IP-SDM		6
**3. Behaviour (external factors)**		
3.1 Factors associated with patients		
3.1.1 Patients' preferences	4	
3.1.2 Patients' culture and values	1	
3.2 Factors associated with IP-SDM as an innovation		
3.2.1 IP-SDM cannot/can be tried on an experimental basis		4
3.2.2 IP-SDM is complex/easy to use	1	
3.3 Factors associated with the environment		
3.3.1 IP-SDM is time-intensive/saves time	15	4
3.3.1.1 IP team members' schedule too full/regularly scheduled IP team meetings	7	3
3.3.1.2 IP-SDM requires the practitioner to choose among tasks	1	
3.3.1.3 Intervention time too short/sufficient to apply IP-SDM without harming patient's health	3	
3.3.2 Insufficient/sufficient resources to apply IP-SDM	10	5
3.3.2.1 Insufficient/sufficient technological and information resources to apply IP-SDM		4
3.3.3 Insufficient/sufficient access to services necessary to apply IP-SDM	1	
3.3.4 Lack of reimbursement/reimbursement for applying IP-SDM	5	1
3.3.5 Ethical issues (confidentiality of patient data, risk of malpractice suits)	3	1
3.3.6 Imbalance/balance of power between health professionals and patients	1	
3.3.7 Geographical location of team members (different locations/proximity)	3	2
**4.Organization**		
4.1 General organizational constraints/facilitators	3	
4.2 Organizational structures and routines	8	4
4.2.1 Different working schedules	2	
4.3 High/low implementation costs	4	1
4.4 Insufficient/sufficient support from the organization	2	5
4.5 Unfavourable/favourable paradigms in the organization	1	1
4.6 Lack of responsiveness/responsiveness by the organization	3	6
4.7 Ministerial unwillingness/willingness		1
4.8 Approach not embedded/embedded within the organization		2
4.9 No leaders/leaders within the organization		1
4.10 Unfavourable/favourable legislation		1
4.11 Revised accreditation standards		1
**5.Barriers/facilitators associated with IP collaboration**		
**5.1 Division of labour**		
5.1.1 Protecting fields of expertise	1	
5.1.2 Practicing in silos	6	
5.1.3 Lacking/sharing knowledge of different disciplinary frameworks	4	7
5.1.4 Disagreeing/agreeing over roles and responsibilities	5	
5.1.5 Sharing responsibilities increases/decreases the work		4
5.1.6 IP-SDM uses professionals' skills and strengths inefficiently/efficiently	1	
**5.2 Interactions**		
5.2.1 Lack of effective communication/effective communication	2	1
5.2.2 Lack of shared working methods/shared working methods	1	
5.2.3 Lack of/presence of a shared health care philosophy regarding patients' needs		1
5.2.4 Interpersonal incompatibility/compatibility	1	
5.2.5 Imbalance/balance of power between professionals	9	2
5.2.6 Lack of trust/trust	4	5
5.2.7 Lack of respect/respect		4
5.2.8 Lack of/presence of team cohesion (appreciation of others' contributions)	1	3
5.2.9 Lack of/presence of continuous interactions	2	
**5.3 Environment**		
5.3.1 Unstable teams (movement of staff)/stable teams	4	3

IP-SDM, inter-professional shared decision making.

The three most common organizational barriers were organizational routines, the costs of implementation and the organization's lack of responsiveness to the IP-SDM model. The three most often reported organizational facilitators were the organization's responsiveness to the model, support by the organization and pre-existing organizational routines consistent with IP and/or SDM.

### Participants' critical appraisal of the model

Most participants agreed or strongly agreed that the IP-SDM model was logical (73.4%), testable (62.0%), and relevant to SDM (83.5%), inter-professionalism (77.2%) and primary care (59.5%). More than half also agreed or strongly agreed that the schematic representation of the model was clear (55.7%). Fewer participants agreed or strongly agreed that the concepts were clear (50.6%), that the relationships between the concepts were clear (46.8%), and that they would be willing to test the model in a clinical setting (40.5%). Results did not vary significantly by the level of the health care system (micro, meso, macro) that participants represented (*P* > 0.05) or by participants' gender (*P* > 0.05). Also, there were no significant differences associated with the interviewers (*n* = 3) who led the interviews (individual and group) (*P* > 0.05). Based on results from the theory appraisal, in particular participants' feedback that the graphical presentation of the model was complicated and difficult to understand, we revised the model and developed a companion document that defines the concepts used in the model and makes the relational statements between them explicit (document available upon request).

## Discussion

Our IP-SDM model for primary care was developed using a comprehensive process that included theory analysis and group consensus methods and has been reviewed by key stakeholders from three levels of the health care system. Overall, it was positively received though less than half of the participants agreed or strongly agreed that its concepts and the relationships were clear. Participants found the model to be logical, testable and relevant to SDM, inter-professionalism and useful in primary care. They proposed changes that were reviewed by team members and integrated in a revised model. They also identified barriers and facilitators to implementing the model in clinical practice. Participants' suggestions to improve the clarity of the model included enlarging the concept of family to include significant others, changing the term ‘equipoise’ and clarifying types of outcomes.

Very few conceptual models are designed with the approach we have detailed here. In proposing new conceptual models, developers usually only refer to the literature and their own expert judgment. However, potential users' assessment of a model can provide important insight on how an initial model designed from a literature review and expert opinion should be modified to better guide clinical practice. Accordingly, we had 79 participants assess the validity of our model in order to apply it in clinical practice. This was very useful given that some of participants' proposed changes reinforced what we had intended to accomplish (e.g. put the patient at the centre of the process) but had not yet achieved in the earlier version. Our experience thus confirms the great value of adding this layer of feedback to the elaboration of a conceptual model before its implementation.

The participation of 79 individuals in our study also confirmed the assertion of scholars from inter-professionalism [25] and SDM that stakeholders from all levels of the health care system are demonstrating increased interest in these two domains. In addition, our study devised an innovative method of presenting our initial IP-SDM model to action-oriented individuals: the video vignette. Without this solution, it was more difficult to explain how a conceptual model could translate into clinical practice.

Team members accepted most of the changes proposed by participants and revised the model accordingly. Changes that were not accepted often came from only one single individual interview or group interview but were not supported by other research. For example, one group interview suggested we change the term ‘patient’ to the term ‘client,’‘consumer,’ or ‘person facing a decision’. Studies have found, however, that patients want to be called ‘patients’[26]. Another suggestion was to include non-regulated health care providers as IP team members influential in the SDM process. However, we limited the description of individuals involved in SDM to regulated professions because it is easier to identify these individuals and we are planning to work with regulated health care professionals to implement the model within the Canadian health care system. Finally, participants' feedback helped the model better represent the transition between the patient-family team and the IP team. In other words, depending on the setting, patients and family may be more or less integrated into the IP team.

Consistent with the findings of other implementation studies, the most frequently reported barrier to the potential implementation of this IP-SDM conceptual model in clinical practice was time constraints [27,28]. Time, as well as insufficient human, material and/or financial resources were also identified in a recent synthesis of barriers and facilitators influencing implementation of SDM in clinical practice [22]. Indeed, a key condition for a successful collaborative practice is the availability of time to interact and spaces to meet [29]. Although time is considered a barrier, research has shown that engaging patients in decision making does not necessarily produce a statistically significant increase in the time necessary to interact with patients: rather, in SDM, time is employed differently, with more time spent on discussing the decision than on giving information [30–32]. Indeed, some participants felt that sharing responsibilities would rather optimize efficacy and save time.

Another barrier identified in our study was the imbalance of power between the health professionals whom participants considered influential in achieving an IP approach to SDM. This finding is not surprising, given that one of the key elements necessary to achieving IP collaboration in clinical practice is symmetry of power [25]. Actually, equality between professionals is one of the basic characteristics of a collaborative practice; research has shown that collaboration is hindered by power differences based on gender stereotypes and social status [29]. In reality, however, participants reported that the doctor's symbolic authority is still very strong.

Our results are congruent with the literature on inter-professionalism. For example, the most frequently reported facilitator to implementing the initial IP-SDM model was education and training. This is supported by other research that contends training for inter-professionalism is essential [33]. Indeed, the need for adequate training is a common implementation strategy identified in both SDM and IP literature [19,34]. Besides education and training in IP and SDM, the motivation to achieve an IP approach to SDM and mutual knowledge and understanding of disciplinary roles are other facilitators identified in the literature on IP collaboration. Also, patient decision aids may have a role to play in fostering an IP approach to SDM [35,36] as they have been shown to increase the adoption of SDM-related behaviours in health care professionals [37].

Overall, the revised IP-SDM model proposes that the patient and his or her family (including significant others) are a distinct and active part of the SDM team. As such, they collaborate with the IP team throughout the SDM process. The IP team is composed of health care professionals who care for the patient and influence the SDM process through their roles and relationships. Their roles include two unique ones to this model: the initiator of the SDM process and the decision coach. To be effective, the IP team must develop a collaborative relationship with authentic, constructive and honest communication mutual trust and respect among team members as well as between team members and the patient. The team must provide integrated and cohesive care and share power among its members. The team members must be able to exercise their partnership and share their knowledge regularly and without interruptions, communicating information systematically throughout the therapeutic process and using well-designed information and communication technologies. Broader factors are likely to affect the ability of the IP team to collaborate with the patient in decision making. For this reason, the organization will likely need to modify the environment of practice in order to facilitate the implementation of an IP approach. Finally, professional regulatory and institutional standards may need to be adapted to facilitate an IP approach to patient care.

Our study has limitations. First, we used a snowball strategy to identify potential participants. Our findings are therefore dependant on who agreed to participate and do not necessarily represent the perspectives of all stakeholders. Second, only 48% of the participants watched the video. It is therefore possible that those who watched the video understood the initial IP-SDM model differently from those who did not. In other words, not all participants may have responded to the same model and concepts. Our mixed methods study design permitted us to further explore this issue by further examining the results from the nine theory criteria (the quantitative results) that showed no difference between those who watched the video and those who did not.

### Implications for practice, education and future research

Our study fills an important gap in the knowledge about how IP teams can engage patients and patients' significant others in the decision-making process [38]. The revised IP-SDM model stresses the importance of facilitating communication between the individuals involved in various phases of the decision-making process, with a view of sharing knowledge and ultimately developing a common understanding of the issues at stake. It makes explicit the role of a decision coach and family members and includes the principal elements of IP collaboration. Educators of inter-professionalism may want to refer to this model to foster the practice of SDM by IP teams. However, further research is needed to better understand how IP teams collaborate to achieve SDM, determine types of relationships that are essential to IP-SDM processes, and identify interventions to facilitate implementation of an IP approach to SDM in routine clinical practice.

## Conclusion

Our research team drew on health professionals' and other stakeholders' assessment of our new IP-SDM conceptual model to revise the newly proposed model. The revised model merges the micro, meso and macro levels in an integrated version that can help inform an IP approach to SDM in primary care. Future research should focus on how this conceptual model can help health professionals engage patients in SDM as part of an IP team. This research could address the barriers and build upon the facilitators identified in this study.

## References

[1] Kuzel AJ (2009). Ten steps to a patient-centered medical home. Family Practice Management.

[2] Charles C, Gafni A, Whelan T (1997). Shared decision-making in the medical encounter: what does it mean? (or it takes at least two to tango). Social Science and Medicine.

[3] Elwyn G, Edwards A, Kinnersley P (1999). Shared decision-making in primary care: the neglected second half of the consultation. British Journal of General Practice.

[4] Makoul G, Clayman ML (2006). An integrative model of shared decision making in medical encounters. Patient Education and Counseling.

[5] D'Amour D, Ferrada-Videla M, San Martin Rodriguez L, Beaulieu MD (2005). The conceptual basis for interprofessional collaboration: core concepts and theoretical frameworks. Journal of Interprofessional Care.

[6] D'Amour D, Oandasan I (2005). Interprofessionality as the field of interprofessional practice and interprofessional education: an emerging concept. Journal of Interprofessional Care.

[7] Oandasan I (2007). Teamwork and healthy workplaces: strengthening the links for deliberation and action through research and policy. Healthcare Papers.

[8] Xyrichis A, Ream E (2008). Teamwork: a concept analysis. Journal of Advenced Nursing.

[9] Montori VM, Gafni A, Charles C (2006). A shared treatment decision-making approach between patients with chronic conditions and their clinicians: the case of diabetes. Health Expectations.

[10] Stacey D, Legare F, Pouliot S, Kryworuchko J, Dunn S (2010). Shared decision making models to inform an interprofessional perspective on decision making: a theory analysis. Patient Education and Counseling.

[11] Haggerty JL, Reid RJ, Freeman GK, Starfield BH, Adair CE, McKendry R (2003). Continuity of care: a multidisciplinary review. British Medical Journal.

[12] Wennberg JE, O'Connor AM, Collins ED, Weinstein JN (2007). Extending the P4P agenda, part 1: how medicare can improve patient decision making and reduce unnecessary care. Health Affairs.

[13] Campbell M, Fitzpatrick R, Haines A, Kinmonth AL, Sandercock P, Spiegelhalter D, Tyrer P (2000). Framework for design and evaluation of complex interventions to improve health. British Medical Journal.

[14] Brink-Huis A, van Achterberg T, Schoonhoven L (2008). Pain management: a review of organisation models with integrated processes for the management of pain in adult cancer patients. Journal of Clinical Nursing.

[15] Jonas WB, Beckner W, Coulter I (2006). Proposal for an integrated evaluation model for the study of whole systems health care in cancer. Integrative Cancer Therapies.

[16] Légaré F, Stacey D, Gauvin FP (2010). Interprofessionalism and shared decision-making in primary care: a stepwise approach towards a new model. Journal of Interprofessional Care.

[17] Legare F, Stacey D, Graham ID (2008). Advancing theories, models and measurement for an interprofessional approach to shared decision making in primary care: a study protocol. BMC Health Services Research.

[18] Patton MQ (1990). Qualitative Evaluation and Research Methods.

[19] Gagnon S, Labrecque M, Njoya M, Rousseau F, St-Jacques S, Legare F (2010). How much do family physicians involve pregnant women in decisions about prenatal screening for Down syndrome?. Prenatal Diagnosis.

[20] Fawcett J, Fawcett J, Fithian M (1989). Conceptual models and theories. Analysis and Evaluation of Conceptual Models of Nursing.

[21] Walker L, Avant K (2005). Strategies for Theory Construction in Nursing.

[22] Légaré F, Ratté S, Gravel K, Graham ID (2008). Barriers and facilitators to implementing shared decision-making in clinical practice: update of a systematic review of health professionnals' perceptions. Patient Education and Counselling.

[23] Pluye P, Potvin L, Denis JL, Pelletier J (2004). Program sustainability: focus on organizational routines. Health Promotion International.

[24] Elwyn G, Edwards A, Kinnersley P, Grol R (2000). Shared decision making and the concept of equipoise: the competences of involving patients in healthcare choices. British Journal of General Practice.

[25] Gaboury I, Bujold M, Boon H, Moher D (2009). Interprofessional collaboration within Canadian integrative healthcare clinics: key components. Social Science and Medicine.

[26] Deber RB, Kraetschmer N, Urowitz S, Sharpe N (2005). Patient, consumer, client, or customer: what do people want to be called?. Health Expectations.

[27] Gagnon MP, Legare F, Labrecque M, Fremont P, Cauchon M, Desmartis M (2007). Perceived barriers to completing an e-learning program on evidence-based medicine. Informatics in Primary Care.

[28] van Steenkiste B, van der Weijden T, Stoffers HE, Grol R (2004). Barriers to implementing cardiovascular risk tables in routine general practice. Scandinavian Journal of Primary Health Care.

[29] San Martin-Rodriguez L, Beaulieu MD, D'Amour D, Ferrada-Videla M (2005). The determinants of successful collaboration: a review of theoretical and empirical studies. Journal of Interprofessional Care.

[30] Stacey D, O'Connor AM, Graham ID, Pomey MP (2006). Randomized controlled trial of the effectiveness of an intervention to implement evidence-based patient decision support in a nursing call centre. Journal of Telemedicine and Telecare.

[31] Stacey D, Pomey MP, O'Connor AM, Graham ID (2006). Adoption and sustainability of decision support for patients facing health decisions: an implementation case study in nursing. Implementation Science.

[32] Whelan T, Sawka C, Levine M (2003). Helping patients make informed choices: a randomized trial of a decision aid for adjuvant chemotherapy in node-negative breast cancer. Journal of the National Cancer Institute.

[33] Reeves S, Zwarenstein M, Goldman J, Barr H, Freeth D, Hammick M, Koppel I (2008). Interprofessional education: effects on professional practice and health care outcomes. Cochrane Database of Systematic Reviews.

[34] Ho K, Jarvis-Selinger S, Borduas F (2008). Making interprofessional education work: the strategic roles of the academy. Academic Medicine.

[35] Lalonde L, O'Connor AM, Drake E, Duguay P, Lowensteyn I, Grover SA (2004). Development and preliminary testing of a patient decision aid to assist pharmaceutical care in the prevention of cardiovascular disease. Pharmacotherapy.

[36] Lalonde L, O'Connor AM, Duguay P, Brassard J, Drake E, Grover SA (2006). Evaluation of a decision aid and a personal risk profile in community pharmacy for patients considering options to improve cardiovascular health: the OPTIONS pilot study. International Journal of Pharmacy Practice.

[37] Nannenga MR, Montori VM, Weymiller AJ (2009). A treatment decision aid may increase patient trust in the diabetes specialist. the statin choice randomized trial. Health Expectations.

[38] Towle A, Bainbridge L, Godolphin W, Katz A, Kline C, Lown B, Madularu I, Solomon P, Thistlethwaite J (2010). Active patient involvement in the education of health professionals. Medical Education.

